# Agency in Contraceptive Decision-Making in Patient Care: a Psychometric Measure

**DOI:** 10.1007/s11606-022-07774-0

**Published:** 2022-09-07

**Authors:** Cynthia C. Harper, Lavanya Rao, Isabel Muñoz, Lisa Stern, Jennifer L. Kerns, Miriam Parra, Brittany D. Chambers, Corinne H. Rocca

**Affiliations:** 1 Department of Obstetrics, Gynecology and Reproductive Sciences, University of California, San Francisco, CA USA; 2 Advancing New Standards in Reproductive Health (ANSIRH), University of California, San Francisco, CA USA; 3 Coalition to Expand Contraceptive Access (CECA), San Francisco, CA USA; 4 Department of Human Ecology, University of California, Davis, CA USA

**Keywords:** contraceptive decision-making agency, reproductive autonomy, contraceptive coercion, patient agency, patient-reported outcomes

## Abstract

**Background:**

Patient agency in contraceptive decision-making is an essential component of reproductive autonomy.

**Objective:**

We aimed to develop a psychometrically robust measure of patient contraceptive agency in the clinic visit, as a measure does not yet exist.

**Design:**

For scale development, we generated and field tested 54 questionnaire items, grounded in qualitative research. We used item response theory–based methods to select and evaluate scale items for psychometric performance. We iteratively examined model fit, dimensionality, internal consistency, internal structure validity, and differential item functioning to arrive at a final scale.

**Participants:**

A racially/ethnically diverse sample of 338 individuals, aged 15–34 years, receiving contraceptive care across nine California clinics in 2019–2020.

**Main Measures:**

Contraceptive Agency Scale (CAS) of patient agency in preventive care.

**Key Results:**

Participants were 20.5 mean years, with 36% identifying as Latinx, 26% White, 20% Black, 10% Asian/Native Hawaiian/Pacific Islander. Scale items covered the domains of freedom from coercion, non-judgmental care, and active decision-making, and loaded on to a single factor, with a Cronbach’s α of 0.80. Item responses fit a unidimensional partial credit item response model (weighted mean square statistic within 0.75-1.33 for each item), met criteria for internal structure validity, and showed no meaningful differential item functioning. Most participants expressed high agency in their contraceptive visit (mean score 9.6 out of 14). One-fifth, however, experienced low agency or coercion, with the provider wanting them to use a specific method or to make decisions for them. Agency scores were lowest among Asian/Native Hawaiian/Pacific Islander participants (adjusted coefficient: -1.5 [-2.9, -0.1] vs. White) and among those whose mothers had less than a high school education (adjusted coefficient; -2.1 [-3.3, -0.8] vs. college degree or more).

**Conclusions:**

The Contraceptive Agency Scale can be used in research and clinical care to reinforce non-coercive service provision as a standard of care.

## INTRODUCTION

Agency in contraceptive decision-making is a key component of overall reproductive autonomy, or the ability to make choices about childbearing, pregnancy, and contraception.^1^ Several qualitative studies from the patient perspective have explored how provider bias can show up in contraceptive care, limiting the patient’s ability to make fully voluntary choices.^2–5^ In this study, we define contraceptive agency as the ability and capacity to decide about contraception, without undue influence, judgment, or coercion from healthcare providers. Contraceptive agency is especially important among patients in communities that have experienced reproductive harms, including from racism or contraceptive coercion in the healthcare system.^6–10^

Researchers have noted that to address health equity goals, new conceptual frameworks and metrics are needed to capture patient experiences of bias or coercion where it may impact reproductive health care.^11–13^ A framework for contraceptive autonomy has been recently delineated that considers free voluntary choice, including whether or not to use contraception.^11^ In terms of measures, there have been advances in the development of measures of women’s agency vis à vis sexual partners, but not in the context of clinical care.^1,14–16^ In clinical care, measures of the quality of contraceptive care, the Interpersonal Quality of Family Planning (IQFP) scale, and its shorter version, the PCCC, have been developed and increasingly used, helping to shift contraceptive care toward greater attention to patient preferences and needs.^17,18^ However, there still exists a scientific gap in the measurement of patient agency and freedom from coercion in the clinic visit. This study adds to existing measurement research by addressing this scientific gap and focusing on patient contraceptive agency in interactions with the provider. Our study aim was to develop the Contraceptive Agency Scale (CAS) and evaluate it for validity and reliability within a racially/ethnically diverse sample.

## MATERIALS AND METHODS

This study uses psychometric techniques to evaluate item properties and performance in the construction of a robust measurement instrument for patient contraceptive agency. In the psychometric scale development and analyses presented here, we conducted a field test to evaluate item properties and performance, reducing a set of 54 items into a 7-item scale, with evidence of reliability and validity. We then used multivariable regression analysis to test for differences in contraceptive agency among patients in communities that may have experienced bias in care, including patients of color, LGBTQ+ patients, or those with low socioeconomic status (SES).

### Formative Qualitative Work

Prior to this study, we conducted qualitative work to inform early stages of scale development. We used a multi-step development process, based on community feedback and qualitative research.^19^ We sought community input at the outset from the community advisory board of the University of California, San Francisco Preterm Birth Initiative. Community members provided guidance on study design, proposed study sites, content areas for instruments, and revisions to the topic guides so they related more closely to their experiences as patients.

In our formative work, we delineated our conceptual framework, drawing from principles of patient-centered care, defined by the Institute of Medicine as care that is responsive to patient preferences, needs, and values.^18,20^ We also included concepts of non-coercion and empowerment in our construct of agency from the reproductive justice and gender literature.^10,16^ We explored patient experiences of contraceptive agency in a series of focus groups and in-depth interviews conducted 2017–2019 in three reproductive health facilities in California. The sample of 30 participants included representation from Latinx, Black, White, Asian, and multiracial individuals. A constructivist grounded theory approach was used to analyze the data. Through our review of the literature and formative qualitative work, we identified several domains comprising contraceptive agency, including freedom from coercion, nonjudgmental care, and active decision-making.^21^ We generated candidate items across these domains, drawing perspectives, concepts, and wording from the qualitative data, and tested item comprehension in ten cognitive interviews, simplifying words and refining phrases into relatable items from participant feedback.

### Procedures and Participants

We recruited study participants receiving contraceptive care across nine California clinics in 2019–2020 to complete surveys with the set of items on contraceptive agency and decision-making. Study sites were primarily Department of Health and non-profit community clinics providing primary care and reproductive healthcare. Sites were selected to ensure the scale measure reflected experiences from diverse patient populations and included federally qualified health centers, school-based health centers, reproductive health clinics, and an outpatient public hospital obstetrics and gynecology clinic. Eligibility criteria included individuals aged 15–34 years and assigned female at birth, who spoke and read English or Spanish, were sexually active in the last 6 months, and were receiving contraceptive care. We aimed to recruit over 300 participants, determined to be sufficient to estimate item parameters with reasonably small standard errors.^22,23^

Research assistants recruited participants in clinic waiting rooms. Clinic front office staff informed age-eligible patients about the study. Research assistants inquired if the patient was interested and, if so, described the study, screened for eligibility, answered questions, and obtained electronic informed consent on a tablet. After their clinic visit, the participants completed a self-administered questionnaire on the tablet. Surveys included 54 items related to contraceptive agency and decision-making during the clinic visit, such as “My providers helped me to choose a method of birth control that could work for me” and “My provider wanted to make my birth control decisions for me.” Items had Likert scale answer categories: strongly agree, agree, neither agree nor disagree, disagree, strongly disagree, or does not apply (coded as missing). We collected data on socioeconomic and reproductive health factors. Surveys took approximately 20 min to complete. The participants received remuneration of $20 cash or gift card. The study was approved by the Institutional Review Board of the University of California, San Francisco.

### Analyses

We employed both the item response theory (IRT) and classical test theory methods to iteratively examine item performance and reduce the item set toward a final measure.^19,24^ IRT is a methodology from measurement science used to develop and measure latent constructs.^22,25^ It offers advantages over traditional scale evaluation methods, including a broader tool set for examining item performance, flexibility to allow the “distance” between response categories to vary, and capacity to incorporate external variables (socio-demographics) directly into measurement models to assess differential performance of items.^26,27^ IRT uses item responses to fit a logistic random intercept model and create a linear (logit) scale representing measured characteristics. Recently, IRT has begun to be applied to develop rigorous reproductive health measures of latent constructs.^28–30^

To reduce the item set and select final items, we first assessed item acceptability, removing those with > 5% missing or “Does not apply.” We examined the distribution of responses on items to make sure that they accurately captured the different levels of the underlying construct and served to differentiate patients’ levels of agency. There was overall low endorsement of categories indicating lower agency, which we anticipated from prior contraceptive research showing positive feelings about care quality.^18^ We therefore collapsed the three lowest response categories in analyses for parsimony (i.e., strongly disagree, disagree, or neither). We also removed items with any resulting category receiving < 5% endorsement, as they did little to differentiate participants’ levels of the underlying construct of agency.^31^

We iteratively fit item responses to a partial credit item response model and examined item fit, dimensionality, internal structure validity, and differential item functioning, removing less optimally performing items until we arrived at 7 final items using the ACER ConQuest software.^32^ We assessed fit-of-item responses to the unidimensional model using the weighted mean-squared index, using the range of 0.75–1.33 as indicating good fit.^33^ We examined internal structure, ensuring that for each item, participants endorsing higher, or more positive, response categories had correspondingly higher overall scale scores. We also generated Wright Maps, plotting item thresholds relative to participant agency levels, to confirm the ordering of each item’s category locations and to ensure items served to differentiate respondents along the spectrum of agency. At all stages of item reduction, we considered the conceptual territory items covered and maintained a final set of items covering a range of domains of agency.

When the final 7 items were selected, we reanalyzed the data to establish the scale’s psychometric properties. In addition to repeating the steps outlined above, we assessed internal consistency with the separation reliability coefficient. To investigate differential item functioning (DIF) between participants, we fit new partial credit DIF models—separately by characteristics—which incorporated item-by-characteristic interaction terms.^34^ The characteristics included age, parity, sexual orientation, race/ethnicity, and maternal education level as an indication of socioeconomic status (SES). We used maternal education as a socioeconomic indicator rather than the participants’ highest educational level because over half of the sample were adolescents and still in high school. Maternal educational level is a useful SES indicator in such cases, as household income is also generally unknown to adolescent participants. We considered item-by-characteristic parameter effect sizes of ≥ 0.6 logits as evidence of DIF.^35,36^

We translated scale properties into a classical framework by summing raw scores across items and examining internal consistency (Cronbach’s *α*), calculating item-total correlations, and ensuring items loaded onto a single factor with an eigenvalue > 1. We imputed values on missing items based on average scores across the other items for participants who had responses to greater than half (4 of 7) of the items.

Although no instruments to measure contraceptive agency exist, we used multivariable regression to investigate variations in contraceptive agency by participant characteristics we hypothesized might reflect structural inequities or provider biases, including race/ethnicity, maternal education, age group, or sexual orientation. These factors do not arise within themselves, but are embedded in structural and social determinants of health.^9^ We used Stata 16.0 for regression analyses (College Station, TX). Finally, we used a Wright Map and tools available in IRT to identify an empirical cut-point for low Contraceptive Agency Scale,^37,38^ and repeated regression analyses using logistic regression.

## RESULTS

There were 338 participants, with a mean age of 20.5 years (Table 1). Fifty-three percent were adolescents (15–19 years). Over one-third (36%) identified as Latinx (a gender-inclusive term); 26% as White; 20% as Black; 10% as Asian, Native Hawaiian, or Pacific Islander (A/NH/PI); and 8% as multiracial or other. Sixteen percent of Latinx participants completed the study in Spanish. Most participants, 86%, reported their mothers had educational levels less than college degree, with 37% less than high school. Eighty-three percent of the participants reported they were heterosexual, 15% bisexual, and 1% each gay/lesbian or other. All reported they were cis-gendered. About one-fifth (21%) had children; 85% reported sex in the past month. Twenty-two percent were not using a contraceptive method, while 20% were using condoms, 16% injectables, 15% oral contraceptive pills, 10% implant, 6% IUD, and 5% vaginal ring or transdermal patch.

**Table 1:** Respondent Characteristics (*n* = 338)

	*n*	%
Age, mean years, SD (range: 15–33) (*n* = 337)	20.5	4.6
Age group (*n* = 337)		
15–19	180	53.4
20–24	78	23.2
25–34	79	23.4
Race/ethnicity		
Latinx	123	36.4
White	87	25.7
Black	66	19.5
Asian, Native Hawaiian, Pacific Islander	34	10.1
Multiracial or other	28	8.3
Maternal education (*n* = 334)		
Less than high school	123	36.8
High school, GED, vocational, some college	165	49.4
College degree, 4-year or more	46	13.8
Parity (*n* = 334)		
0	264	79.0
1	40	12.0
2 or more	30	9.0
Married	28	8.3
Has a main partner	282	83.4
Had sexual intercourse in the last month (*n* = 332)	282	84.9
Sexual orientation (*n* = 337)		
Heterosexual	281	83.4
Bisexual	50	14.8
Gay/lesbian	3	0.9
Pansexual or other	3	0.9
Primary reason for clinic visit		
Contraceptive care	191	56.5
STI testing	67	19.8
Pregnancy test, pre/postnatal	40	11.8
Annual, illness, non-reproductive, other	40	11.8
Current contraceptive method		
None	73	21.6
Withdrawal or other*	24	7.1
Condom	67	19.8
Vaginal ring or transdermal patch	16	4.7
Oral contraceptive pill	50	14.8
Depo-Provera (injection)	55	16.3
Implant	32	9.5
IUD	21	6.2

*Withdrawal *n* = 20, fertility awareness method *n* = 1, emergency contraception *n* = 3

**Fig. 1:**
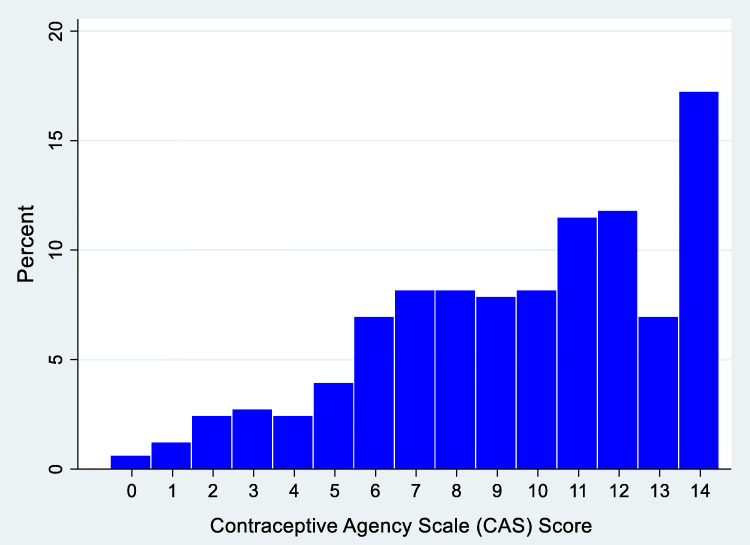
Histogram of CAS responses.

The Contraceptive Agency Scale (CAS) includes both positive and negative items, falling across the domains of freedom from coercion, non-judgmental care, and active decision-making (Table 2). Overall, the participants reported that their providers had facilitated high levels of agency in their contraceptive visit, as shown in the set of scale items. However, negative items revealed patients experienced coercion with the provider making them use a specific method or making decisions for them. As a scale, the distribution of CAS scores—comprised of raw summed scores across the 7 final items (scale range from low to high agency: 0–14)—were left skewed, reflecting the high scores (median = 10, IQR = 7–12) (Fig. 1). CAS items loaded on to a single factor with an eigenvalue > 1, and item-total correlations ranged from 0.64 to 0.75, with a Cronbach’s *α* of 0.80 (Table 3).

**Table 2:** Contraceptive Agency Scale (CAS) Items

	*n*	%
My provider would be open to me trying different birth control methods.^a^ (+)		
Strongly agree	191	56.2
Agree	98	29.4
Neither agree nor disagree	28	8.5
Disagree	7	2.1
Strongly disagree	0	0
I feel that my provider would support me if I wanted to stop using birth control.^b^ (+)		
Strongly agree	198	58.9
Agree	92	27.4
Neither agree nor disagree	31	9.2
Disagree	7	2.1
Strongly disagree	1	0.3
My provider helped me choose a birth control method that could work for me.^c^ (+)		
Strongly agree	172	51.3
Agree	99	29.6
Neither agree nor disagree	38	11.3
Disagree	10	3.0
Strongly disagree	0	0
I felt that my provider made me use a specific birth control method.^b^ (–)		
Strongly disagree	136	40.5
Disagree	74	22.0
Neither agree nor disagree	40	11.9
Agree	22	6.6
Strongly agree	46	13.7
My provider made me feel like I had to use birth control.^b^ (–)		
Strongly disagree	126	37.4
Disagree	109	32.3
Neither agree nor disagree	41	12.2
Agree	18	5.3
Strongly agree	28	8.3
My provider wanted to make my birth control decisions for me.^c^ (–)		
Strongly disagree	184	54.8
Disagree	81	24.1
Neither agree nor disagree	21	6.3
Agree	13	3.9
Strongly agree	14	4.2
I felt that my provider judged me for my birth control decisions.^a^ (–)		
Strongly disagree	206	61.1
Disagree	81	24.0
Neither agree nor disagree	20	5.9
Agree	4	1.2
Strongly agree	9	2.7

(+) item coded “Strongly disagree, disagree, or neither” = 0, “Agree” = 1, “Strongly agree” = 2. (–): item coded “Strongly agree, agree, or neither” = 0, “Disagree” = 1, “Strongly disagree” = 2. Domains: a = non-judgmental care; b = freedom from coercion; c = active decision-making. Does not apply coded as missing

**Table 3:** Contraceptive Agency Scale Reliability and Item Properties

	Classical test (Cronbach’s *α*: 0.80)		Item response model	
	Item-total correl.	Factor loading	Model fit	Difficulty
P open to trying different methods^a^ (+)	0.65	0.58	1.03	− 0.37
P would support stopping^b^ (+)	0.64	0.56	1.09	− 0.33
P helped choose method for me^c^ (+)	0.64	0.56	1.09	− 0.07
P made me use specific method^b^ (–)	0.65	0.54	1.15	0.78
P made me feel had to use^b^ (–)	0.75	0.68	0.93	0.64
P wanted to make decision for me^c^ (–)	0.71	0.66	0.98	− 0.14
P judged me for my decision^a^ (–)	0.72	0.68	0.89	− 0.51

Domains: a = non-judgmental care; b = freedom from coercion; c = active decision-making. Item fit and difficulty are from a unidimensional partial credit item response model for polytomous items. Item fit is the weighted mean-squared fit *t* statistic. Item location is the difficulty parameter in logits

*P* provider

**Fig. 2:**
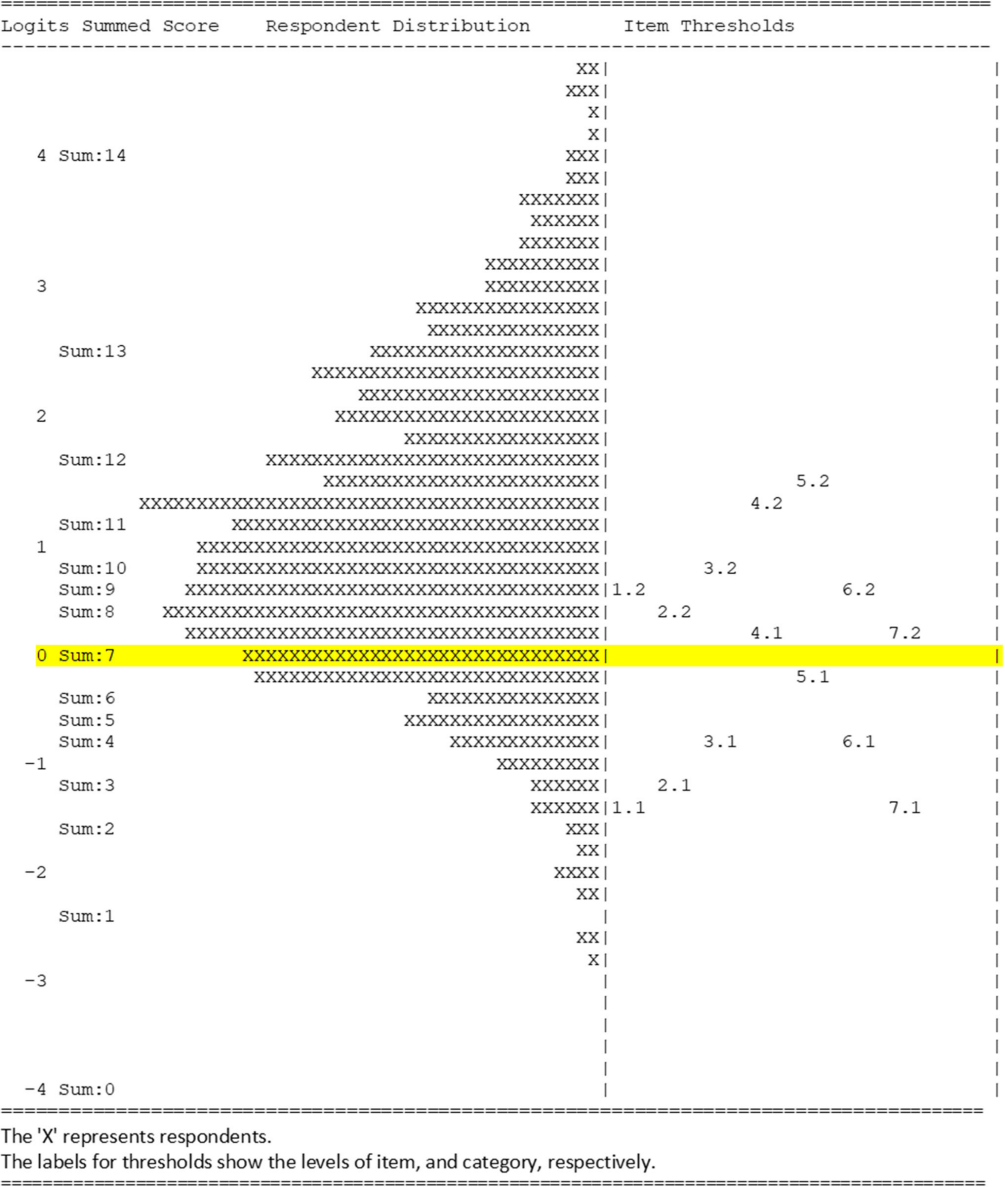
Wright map of latent respondent distribution and Contraceptive Agency Scale item thresholds.

Items fit the unidimensional partial credit item response model (weighed mean square fit statistics ranging from 0.93 to 1.15) and had a person separation reliability of 0.58. Items met all criteria for internal structure validity, with each item having response categories that corresponded to participant CAS scores overall, and item parameters generally covering participant agency levels (Fig. 2). When testing differential item functioning (DIF) separately for each sociodemographic characteristic, there was some evidence of DIF by race/ethnicity and age for one of the 7 scale items. We detected no DIF for any item by maternal education, sexual orientation, or parity, indicating individual item parameters were similar across participants.

After assessing individual item’s performance and scale psychometrics, we examined overall differences in CAS scores for different patient groups. We tested for variations in CAS by characteristics that might reflect provider bias or structural inequities including race/ethnicity, education, age, or sexual orientation (Table 4). CAS scores were lower among participants with lower maternal education. Multivariable regression results showed participants whose mothers had less than a high school education had significantly lower CAS scores (mean 9.0) (*aβ* = 2.1 [0.8, 3.3], *p* ≤ 0.001) than those whose mothers had a college degree or higher (mean 11.1). CAS scores also differed by participant race/ethnicity: Asian/NH/PI (mean 8.8), Latinx (mean 9.3), Black (mean 9.9), and White (mean 10.1). Multivariable regression showed scores among Asian/NH/PI participants were significantly lower from White (mean = 10.1, *p* < 0.05) and Black (mean 9.9, *p* < 0.05) participants. CAS scores did not differ by age group or sexual orientation.

**Table 4:** Contraceptive Agency Scale Mean Scores by Participant Characteristics, and *β* Coefficients from Multivariable Linear Regression Model Predicting CAS (*n* = 322)

	Mean score (SD)	Bivariable models^a^ *β* coefficient (95% CI)	Multivariable model^a^ *aβ* coefficient (95% CI)
Total score, *range 0–14*	9.6 (3.5)		
Age group			
15–19 (reference)	9.9 (3.4)		
20–24	9.4 (3.8)	− 0.37 (− 1.53, 0.78)	− 0.52 (− 1.69, 0.65)
25–34	9.0 (3.6)	− 0.72 (− 1.86, 0.42)	− 0.69 (− 1.92, 0.54)
Race/ethnicity			
Latinx	9.3 (3.7)	− 0.69 (− 1.67, 0.29)	− 0.14 (− 1.17, 0.88)
White (reference)	10.1 (3.2)		
Black	9.9 (3.5)	− 0.10 (− 1.25, 1.04)	0.18 (− 0.97, 1.32)
A/NH/PI	8.8 (3.7)	− 1.26 (− 2.67, 0.15)	− 1.51 (− 2.91, − 0.11)^*†^
Multiracial/other	9.7 (3.7)	− 0.34 (− 1.83, 1.15)	− 0.29 (− 1.75, 1.18)
Maternal education			
< High school	9.0 (3.8)	− 1.88 (− 3.08, − 0.69)^**^	− 2.08 (− 3.34, − 0.82)^***^
High school, GED, vocational, associate’s	9.7 (3.4)	− 1.26 (− 2.04, − 0.12)^*^	− 1.56 (− 2.74, − 0.38)^**^
College degree or more (reference)	11.1 (2.7)		
Sexual orientation			
Heterosexual (reference)	9.7 (3.4)		
Bisexual, gay/lesbian, pansexual/other	9.2 (4.1)	− 0.69 (− 1.73, 0.35)	− 0.80 (− 1.84, 0.24)
Parity			
0 (reference)	9.8 (3.5)		
1	9.6 (3.7)	0.01 (− 1.25, 1.27)	− 0.03 (− 1.31, 1.26)
2 or more	7.9 (3.6)	− 1.61 (− 3.08, − 0.15)	− 1.57 (− 3.15, 0.01)

***p ≤ 0.001; **p ≤ 0.01; *p ≤ 0.05

^a^Models control for recruitment site

^†^A/NH/PI differs from Black at *p* ≤ 0.05

Examining item threshold locations on the Wright Map (Fig. 2), we identified a cut-point of < 7 on the scale as indicating low agency. One in five participants (20%) fell below this threshold, indicating lower agency at their contraceptive visit.

## DISCUSSION

### Principal Findings

This study developed and rigorously evaluated a new psychometric instrument to capture contraceptive agency, the Contraceptive Agency Scale (CAS). Analyses demonstrated that the CAS items fit a unidimensional model, were internally consistent, had excellent internal structure (monotonicity), and generally functioned non-differentially based on participants’ sociodemographic characteristics. While CAS scores were overall reflective of providers having facilitated high agency during the contraceptive care visit, about one-fifth had CAS scores indicating lower agency. Low patient agency showed the provider wanting the patient to use a specific method or even sometimes the provider making contraceptive decisions for the patient.

We found inequities reflected in CAS scores. Among participants attending publicly funded clinics, including FQHCs and other community clinics, lower-SES participants, as measured by maternal education, had relatively low agency in their decisions. Racial/ethnic disparities were identified, with Asian/Native Hawaiian/Pacific Islander participants having relatively low CAS scores. Contraceptive care delivery needs to better meet the needs and preferences of all patients. These findings indicate an area important to redress in patient care is to prioritize each patient’s voice and preferences in their care plan.^9,39^

Reproductive autonomy and agency over contraception have been frequently neglected historically and in the present day, especially among patients of color.^6,8–10,40,41^ While there has been a long-standing need to prevent coercion and to support patients’ agency, there has also been a notable scientific gap in the conceptualization and measurement of these constructs. Reproductive autonomy encompasses a range of fertility decisions, and recently, measures have been developed to capture autonomy in decision-making in maternity care^42,43^ that can help to move the field forward to improve maternal health in key dimensions. In contraceptive care, the IQFP/PCCC scales measure quality of care, covering domains of interpersonal connection, decision support, and adequate information, and have helped to raise the standards and expectations for person-centered care.^17,18^ Some CAS items, such as one about whether the provider helped to choose a method that could work for the patient, have similarities with the quality-of-care items of taking contraceptive preferences seriously, in that these items put the focus on the patients’ desires, with the provider in a supportive role. The CAS adds an important dimension by focusing on whether a patient feels pressure about using birth control at all, or a specific method, and indeed whether they are making their own decisions. The Contraceptive Agency Scale builds on prior work, providing a tool for both research and clinical care to highlight the importance of agency in reproductive autonomy.

## RESEARCH AND CLINICAL IMPLICATIONS

This scale can be used to evaluate patient agency in contraceptive interventions, for example, to ensure autonomy is maintained in efforts to increase access. CAS also can be used to assess and reinforce agency in clinical services. Addressing provider bias in patient care is now being recognized as important for health outcomes.^44^ Administering the scale periodically after clinic visits would be a low-cost way to yield data for quality improvement of services. Additionally, a scientifically developed measure of agency can help to inform programs and policies of health systems on a larger scale. Without a metric, programmatic focus may primarily rest on other quantifiable measures and goals, such as contraceptive uptake, that can potentially lead to the erosion of patient agency.^45–47^

Future research will be needed to test and potentially adapt the scale for use across different settings.^3^ There is also a wider need for measures of contraceptive agency for postpartum care in the hospital and at the 6-week follow-up visit, as well as for post-abortion care.^3,48–50^

## STRENGTHS AND LIMITATIONS

The use of scientific methods to investigate agency in contraceptive decision-making is important for several reasons. First, it addresses a gap in research and evaluation, and can help to move the field beyond existing measures, such as contraceptive use, which do not capture important domains including freedom from coercion.^51^ Most contraceptive interventions do not measure impact on patients’ decision-making agency, largely because high-quality, theory-based measures have not yet been developed. This study relied on rigorous psychometric techniques from item response theory for instrument development and testing. Additionally, the scale development process was informed from the outset by a community advisory board and patient experiences in qualitative research. Another strength of the research is the potential to improve health equity in clinical care by including study participants from patient populations who have experienced the negative impacts of structural inequities in their lives as well as implicit bias by healthcare providers.^44,52^ Allowing for patient agency over contraceptive decisions is an essential step in addressing structural inequities in healthcare.^9^

This research has limitations. Although our scale was field tested in different types of community clinics including primary and reproductive healthcare, all sites were in one geographic area. Future testing in additional settings and populations is needed to confirm item parameters and assess group differences, which may function differently depending on the larger context. While our sample was racially/ethnically diverse and included patients with low maternal education, future research should explore additional SES measures. Furthermore, testing is needed among transgender and gender non-conforming individuals, as well as patients with medical conditions or disabilities. It is important to consider patient agency in contraceptive care in global health settings as well as in future research.^5,11^ Data collection took place directly following the clinic visit, for accuracy in recall, but potentially incurring social desirability bias. The CAS does not capture all possible aspects of an individual’s agency, but is a clinical care measure, capturing the support given, or not given, by a provider for patient agency. The scale does not measure agency with a partner nor agency required to access care. We found in our qualitative research that patients carry past experiences into their visits and agency over method choice can change over time.^21^ Testing of the scale in a longitudinal study could capture changes over time.

## CONCLUSIONS

Notable advances have occurred in sexual and reproductive health to highlight the importance of person-centered care and patient preferences.^17,53^ This study adds to this growing literature with the development of a Contraceptive Agency Scale, a robust psychometric instrument, that measures patient agency, a key aspect of contraceptive care among underserved patient populations. This tool may help promote patient agency as an expected part of high-quality contraceptive care.
